# Supplementary material for: Physico-Chemical Modifications Affecting the Activity and Stability of Cu-Based Hybrid Catalysts during the Direct Hydrogenation of Carbon Dioxide into Dimethyl-Ether
Source: Materials (Basel). 2022 Nov 4;15(21):7774. doi: 10.3390/ma15217774 (PMC9657723; doi:10.3390/ma15217774)
Supplement: Supplementary file 1 [file materials-15-07774-s001.zip › materials-2007384-supplementary.pdf]

materials

MDPI

Supplementary Materials

Physico-chemical modifications affecting the activity and stability of Cu-based hybrid catalysts during the direct hydrogenation of carbon dioxide into dimethyl-ether

Fabio Salomone [1,*], Giuseppe Bonura [2], Francesco Frusteri [2], Micaela Castellino [1], Marco Fontana [1,3], Angelica Monica Chiodoni [3], Nunzio Russo [1], Raffaele Pirone [1], Samir Bensaid [1,*]

[1] Department of Applied Science and Technology (DISAT), Politecnico di Torino, Corso Duca degli Abruzzi 24, 10129 Turin, Italy;
[2] Consiglio Nazionale delle Ricerche - Istituto di Tecnologie Avanzate per l'Energia "Nicola Giordano" (CNR-ITAE), Via Santa Lucia Sopra Contesse 5, 98126 Messina, Italy;
[3] Istituto Italiano di Tecnologia (IIT), Via Livorno 60, 10144 Turin, Italy;
* Correspondence: fabio.salomone@polito.it (F.S.); samir.bensaid@polito.it (S.B.)

Table S1. Apparent densities of each catalyst in the catalytic bed (particle size range: 250–500 μm).

Sample	Ferrierite	CZZ-ferrierite bifunctional catalysts			
	FER	OX 1:2	OX 2:1	WI 1:2	MIX 1:2
Apparent density, $kg_{cat}\cdot m_{bed}^{-3}$	463 ± 20	639 ± 23	902 ± 45	795 ± 50	579 ± 24

Table S2. Relative atomic concentration calculated from ICP measurements for all the calcined samples.

Sample		Relative atomic concentration (at. %)					
		Cu	Zn	Zr	Al	Si	O [1]
Ferrierite (FER)	Nominal	-	-	-	3.1	30.8	66.2
	Actual	-	-	-	3.7 ± 0.3	30.3 ± 4.9	66.1 ± 4.7
CuZnZr (CZZ)	Nominal	28.6	14.3	4.8	-	-	52.3
	Actual	23.9 ± 0.7	16.7 ± 0.6	3.9 ± 0.5	-	-	55.5 ± 1.6
CZZ/FER OX 1:2	Nominal	5.7	2.9	1.0	2.5	24.6	63.4
	Actual	4.9 ± 0.3	2.9 ± 0.2	0.83 ± 0.02	2.6 ± 0.1	25.1 ± 0.5	63.6 ± 0.5
CZZ/FER OX 2:1	Nominal	14.3	7.1	2.4	1.5	15.4	53.9
	Actual	10.6 ± 0.4	5.8 ± 0.3	1.55 ± 0.03	2.09 ± 0.01	19.1 ± 0.4	60.8 ± 0.5
CZZ/FER WI 1:2	Nominal	5.7	2.9	1.0	2.5	24.6	63.4
	Actual	4.8 ± 0.2	2.5 ± 0.1	0.71 ± 0.02	2.5 ± 0.1	25.6 ± 0.3	63.8 ± 0.4
CZZ-FER MIX 1:2	Nominal	5.7	2.9	1.0	2.5	24.6	63.4
	Actual	4.5 ± 0.6	1.7 ± 0.2	2.6 ± 0.1	3.1 ± 0.2	24.0 ± 1.2	64.1 ± 1.2

[1] The oxygen atomic concentration was calculated considering CuO, ZnO, Al_2O_3, ZrO_2 and SiO_2.

Table S3. Textural properties of calcined and spent catalysts estimated by means of N_2 physisorption.

Sample	State	S_{LANG} [1]	S_{mp} [2]	V_{BJH} [3]	V_{mp} [2]	d_p [4]
		m^2/g_{cat}	m^2/g_{cat}	cm^3_{STP}/g_{cat}	cm^3_{STP}/g_{cat}	nm
Ferrierite (FER)	Calcined	456.8	414.7	0.084	0.140	0.7
CZZ/FER OX 1:2	Calcined	311.1	253.5	0.217	0.079	2.7
	Spent	218.5	173.7	0.170	0.054	3.1
CZZ/FER WI 1:2	Calcined	302.1	254.4	0.143	0.081	1.9
	Spent	233.7	204.2	0.115	0.066	2.0
CZZ-FER MIX 1:2	Calcined	337.0	288.2	0.145	0.093	1.7
	Spent	264.1	228.1	0.106	0.073	1.6
CZZ/FER OX 2:1	Calcined	211.6	148.8	0.207	0.037	3.9
	Spent	145.8	94.4	0.196	0.021	5.4

[1] The specific surface area (S_{LANG}) was calculated according to the Langmuir theory.

[2] The micropore specific surface area (S_{mp}) and the micropore volume (V_{mp}) were estimated using the t-plot method.

[3] The pore volume between 1.7 nm and 300 nm (V_{BJH}) was determined by applying the BJH algorithm to the desorption branch of the isotherm.

[4] The average pore diameter was calculated considering cylindrical pores.

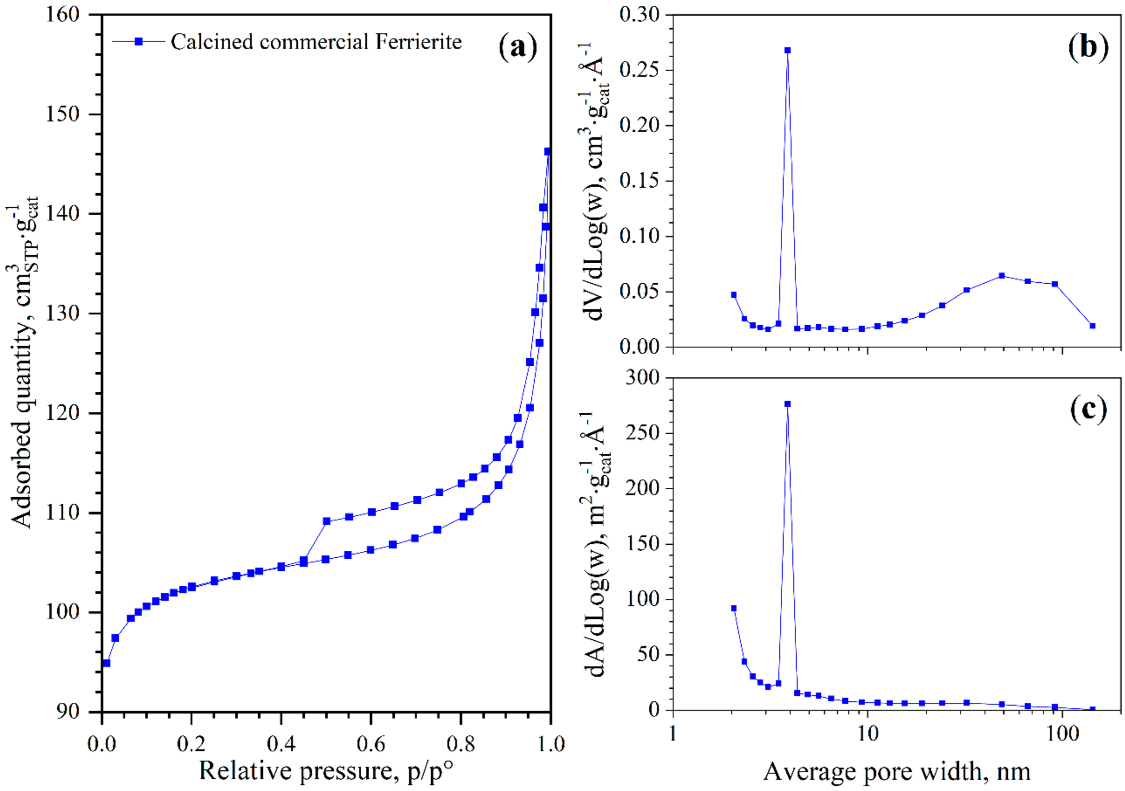

Figure S1. (**a**) N_2-physisorption isotherms, (**b**) pore volume and (**c**) pore area distributions of calcined commercial bare ferrierite.

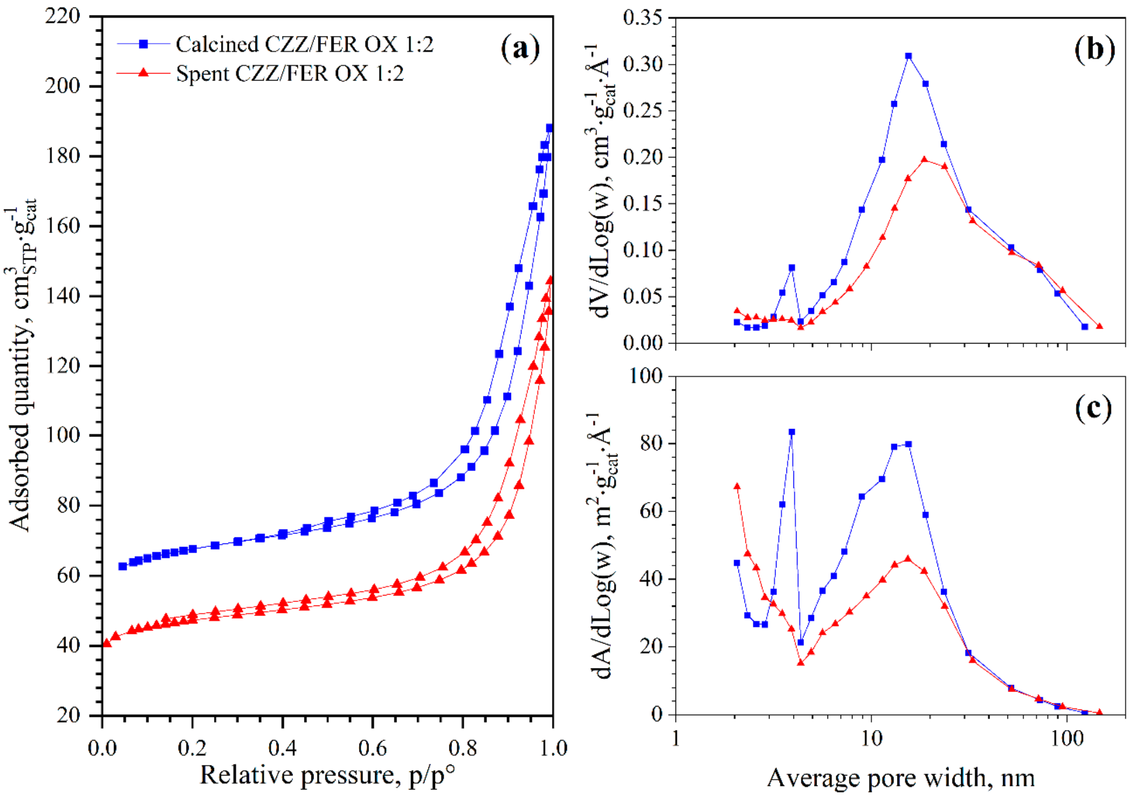

Figure S2. (**a**) N2-physisorption isotherms, (**b**) pore volume and (**c**) pore area distributions of calcined and spent CZZ/FER OX 1:2.

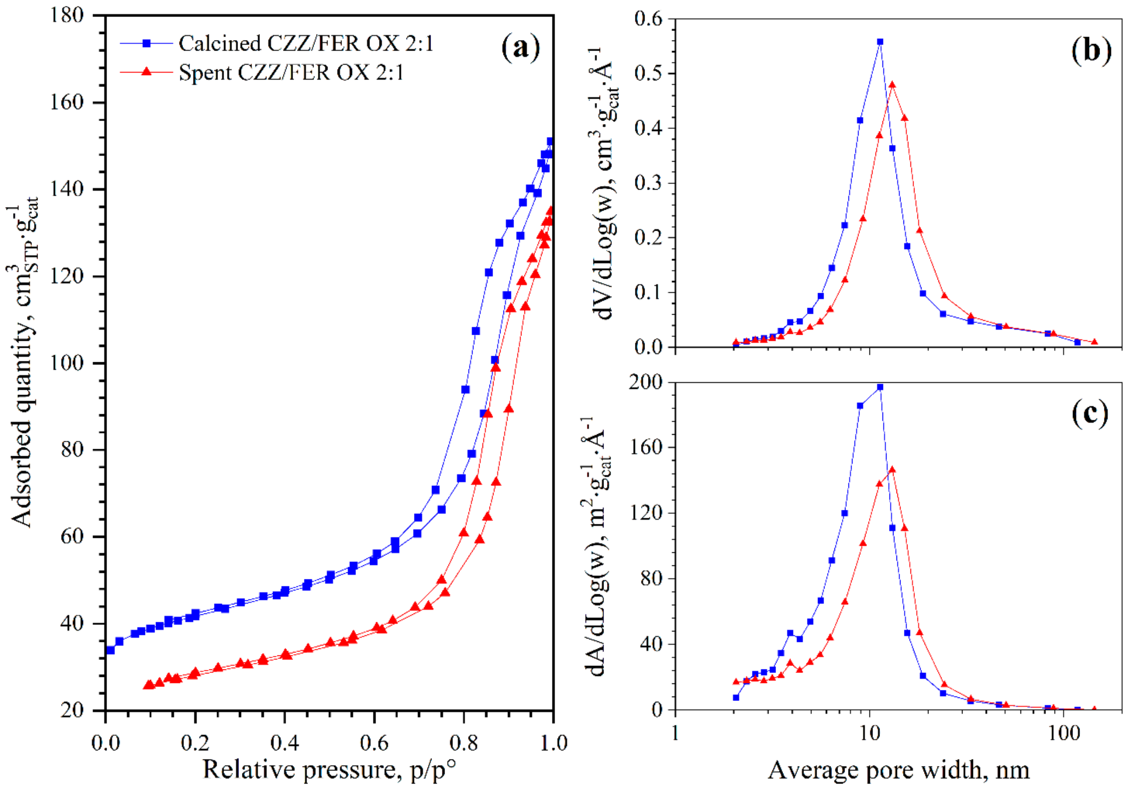

Figure S3. (**a**) N2-physisorption isotherms, (**b**) pore volume and (**c**) pore area distributions of calcined and spent CZZ/FER OX 2:1.

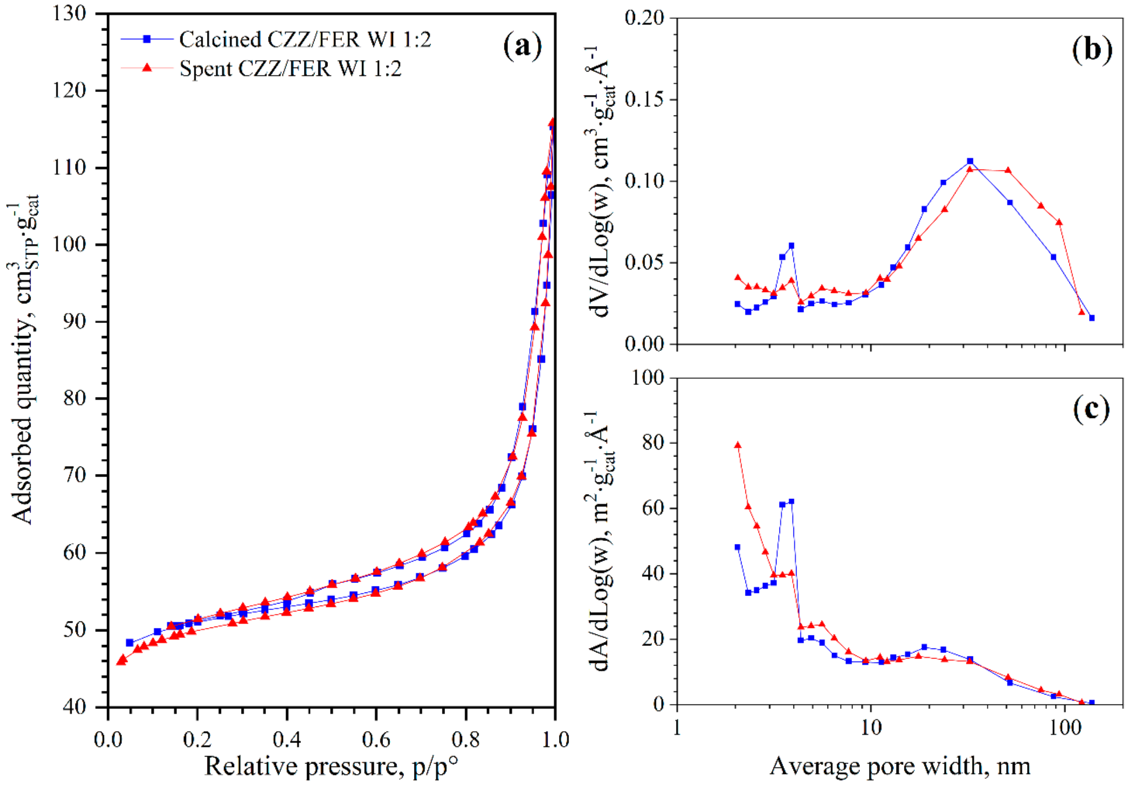

Figure S4. (**a**) N2-physisorption isotherms, (**b**) pore volume and (**c**) pore area distributions of calcined and spent CZZ/FER WI 1:2.

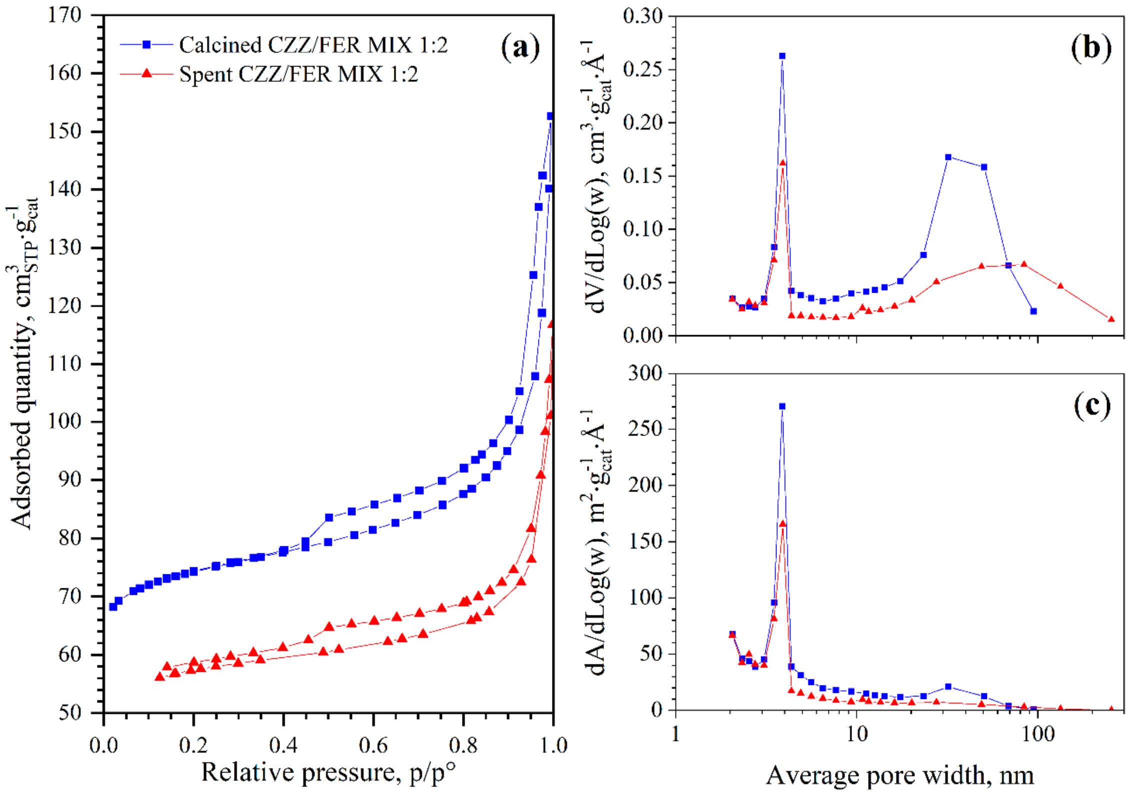

Figure S5. (**a**) N2-physisorption isotherms, (**b**) pore volume and (**c**) pore area distributions of calcined and spent CZZ/FER MIX 1:2.

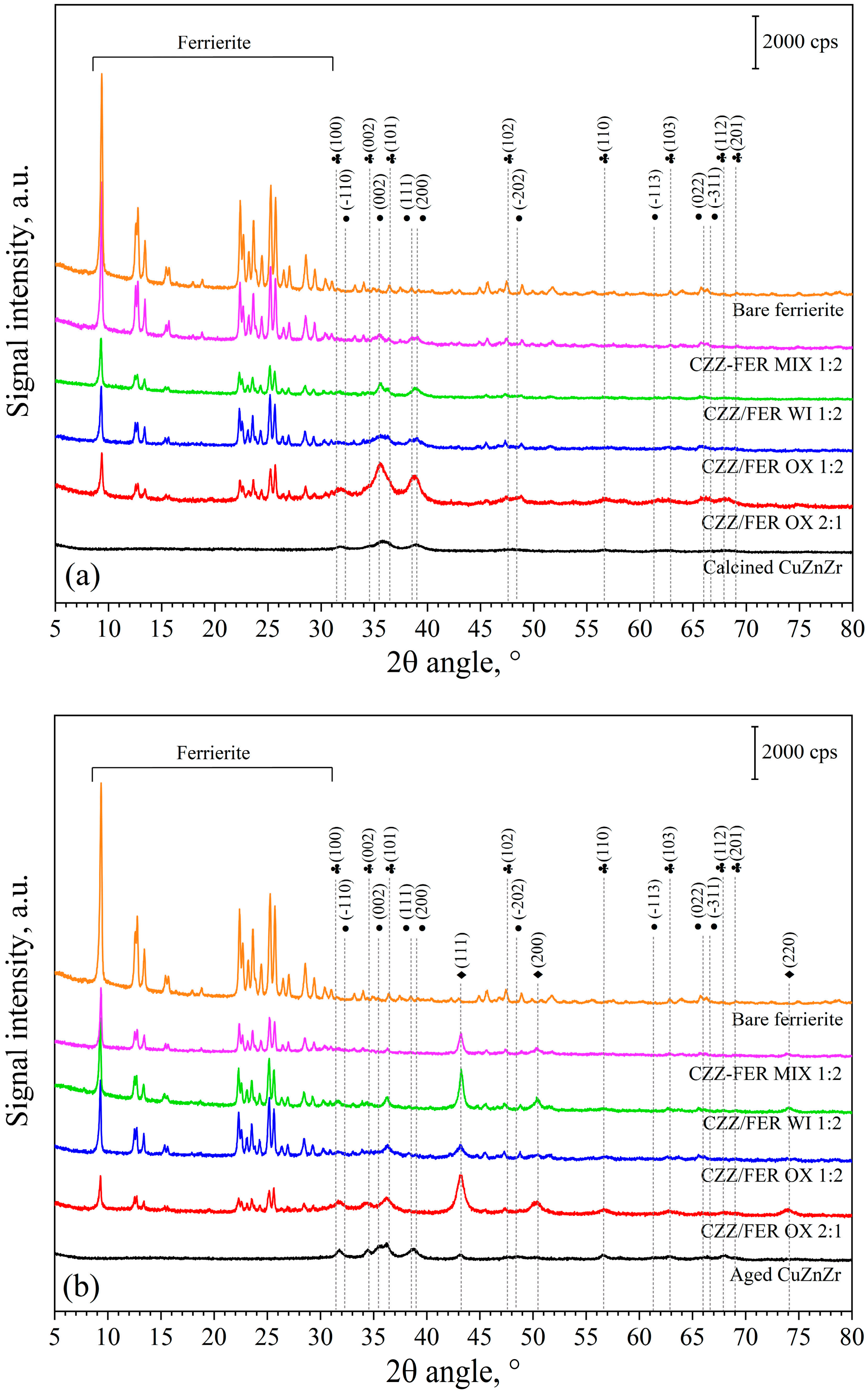

Figure S6. XRD patterns of (**a**) calcined and (**b**)spent catalysts (● CuO, ♦ Cu and ♣ ZnO).

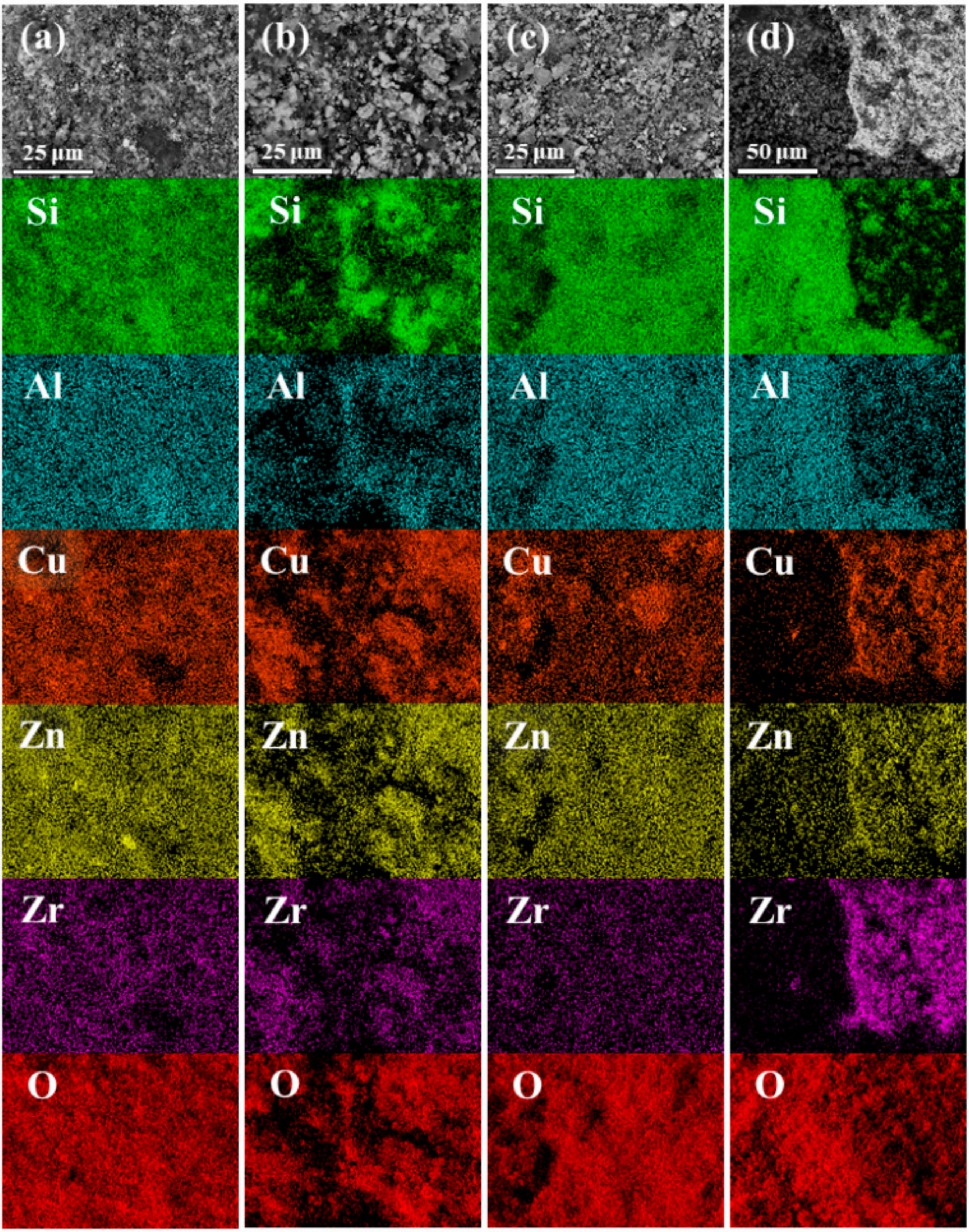

Figure S7. Backscattered FESEM images and EDS maps of calcined (**a**) CZZ/FER OX 1:2, (**b**) CZZ/FER OX 2:1, (**c**) CZZ/FER WI 1:2 and (**d**) CZZ/FER MIX 1:2.

Table S4. Average crystallite size of CuO, Cu and ZnO estimated by means of Scherrer's equation.

Sample	d_{CuO} (nm)		d_{Cu} (nm)	d_{ZnO} (nm)	
	Calcined	Spent	Spent	Calcined	Spent
CuZnZr (CZZ)	8.9	10.5	16.4	7.1	11.9
CZZ/FER OX 2:1	8.2	-	11.6	4.7	8.0
CZZ/FER OX 1:2	5.2	11.2	11.4	5.7	7.0
CZZ/FER WI 1:2	18.5	-	24.9	14.9	16.4
CZZ-FER MIX 1:2	8.8	15.6	24.3	6.2	9.8

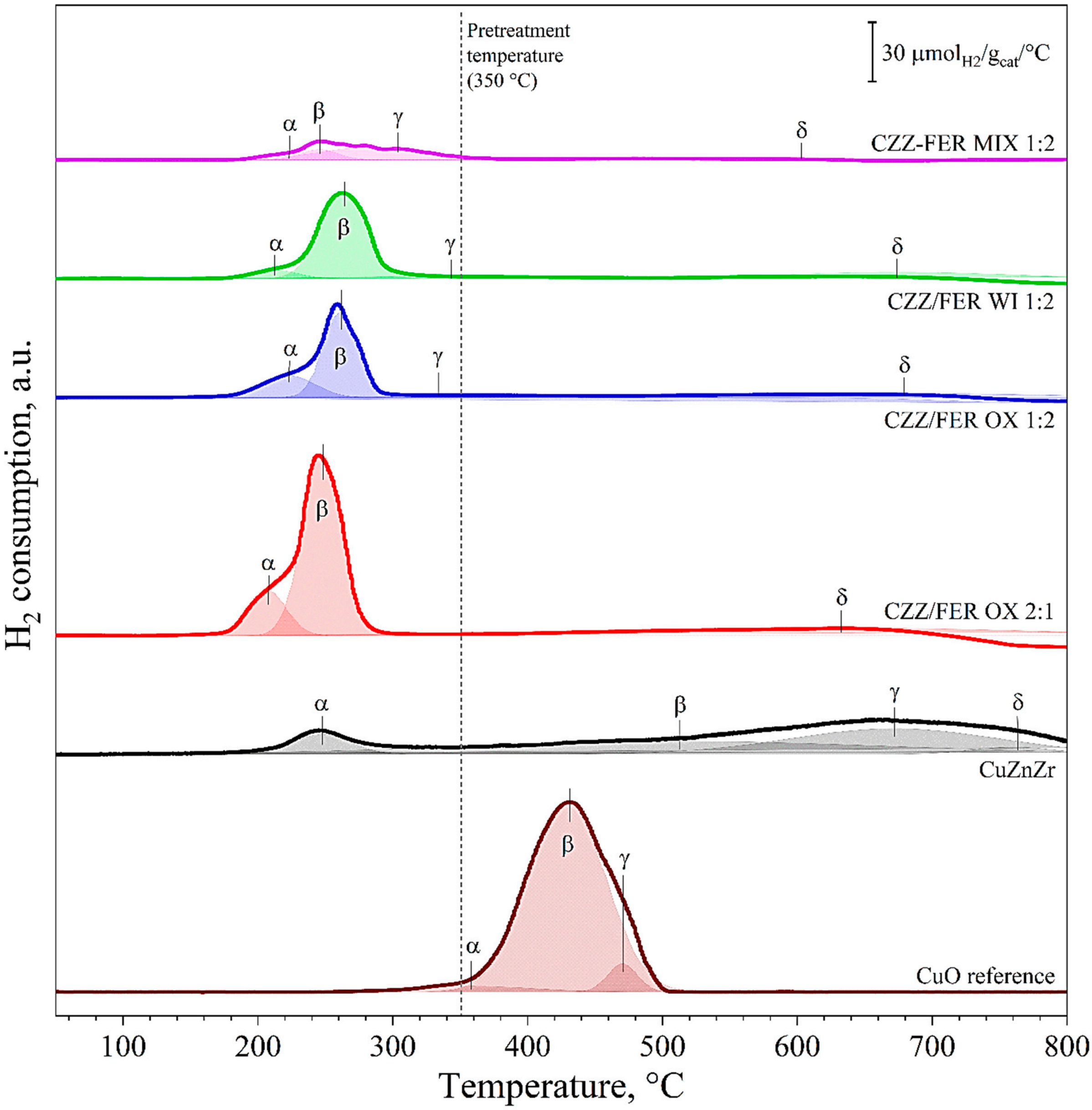

Figure S8. H_2-TPR profiles of the investigated catalysts.

Table S5. Relative atomic concentration (at. %), calculated from high-resolution spectra, for calcined and spent samples measured by XPS.

Sample	State	Relative atomic concentration (at. %)								
		$Cu_{2p3/2}$		$Zn_{2p3/2}$		Zr_{3d}	Si_{2p}		Al_{2s}	O_{1s}
		CuO	Cu_2O and Cu	ZnO	$Zn(OH)_2$	ZrO_2	SiO_2	Si_2O	Al_2O_3	Oxide
Ferrierite (FER)	Calcined	-	-	-	-	-	25.1	-	2.5	59.4
CuZnZr (CZZ)	Calcined	9.5	3.0	5.8	0.5	2.7	-	-	-	31.0
	Spent	11.7	0.9	8.2	0.5	2.4	-	-	-	29.6
CZZ/FER OX 1:2	Calcined	4.9	1.2	3.4	0.9	2.0	15.5	-	-	52.3
	Spent	1.1	3.0	1.8	0.8	1.4	7.3	6.9	-	40.2
CZZ/FER OX 2:1	Calcined	6.6	1.5	5.6	0.7	3.2	8.0	-	-	41.8
	Spent	2.3	2.7	2.4	1.1	1.9	4.6	5.9	-	36.2
CZZ/FER WI 1:2	Calcined	4.8	1.0	4.2	0.8	5.5	8.1	1.1	-	42.3
	Spent	2.0	3.2	2.7	0.3	3.6	5.6	3.0	-	35.1

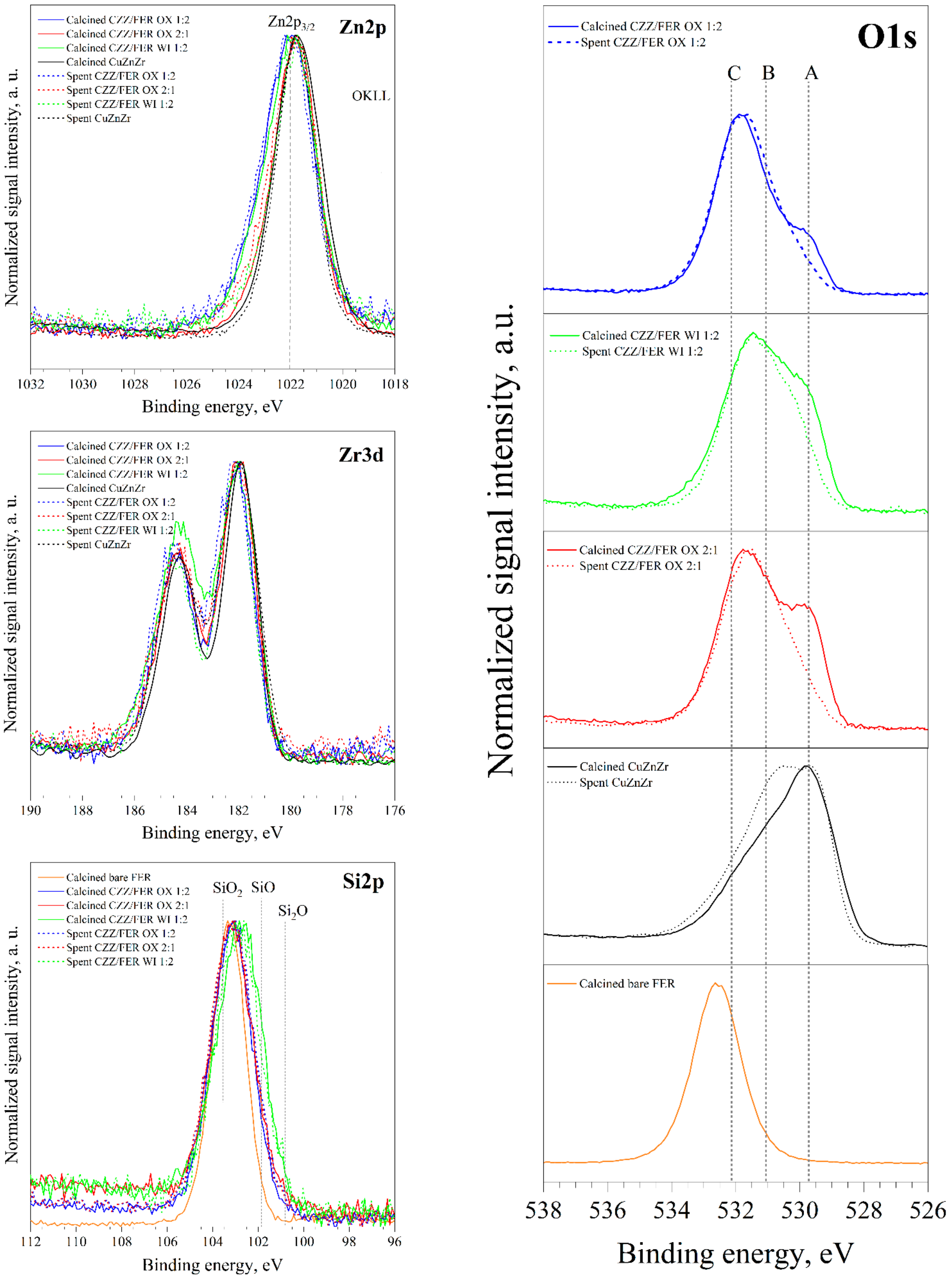

Figure S9. XPS HR spectra for Zn2p3/2, Zr3d, Si2p and O1s peaks of CZZ/FER hybrid samples.

Table S6. Deactivation parameters of the CuZnZr ferrierite-based hybrid catalysts.

Sample	Deactivation constant (h^{-1})	Initial CO_2 conversion (%)
CZZ/FER OX 2:1	$3.63 \cdot 10^{-3}$	19.96
CZZ/FER OX 1:2	$8.75 \cdot 10^{-3}$	7.47
CZZ/FER WI 1:2	$9.36 \cdot 10^{-3}$	9.67
CZZ-FER MIX 1:2	$1.17 \cdot 10^{-3}$	9.00

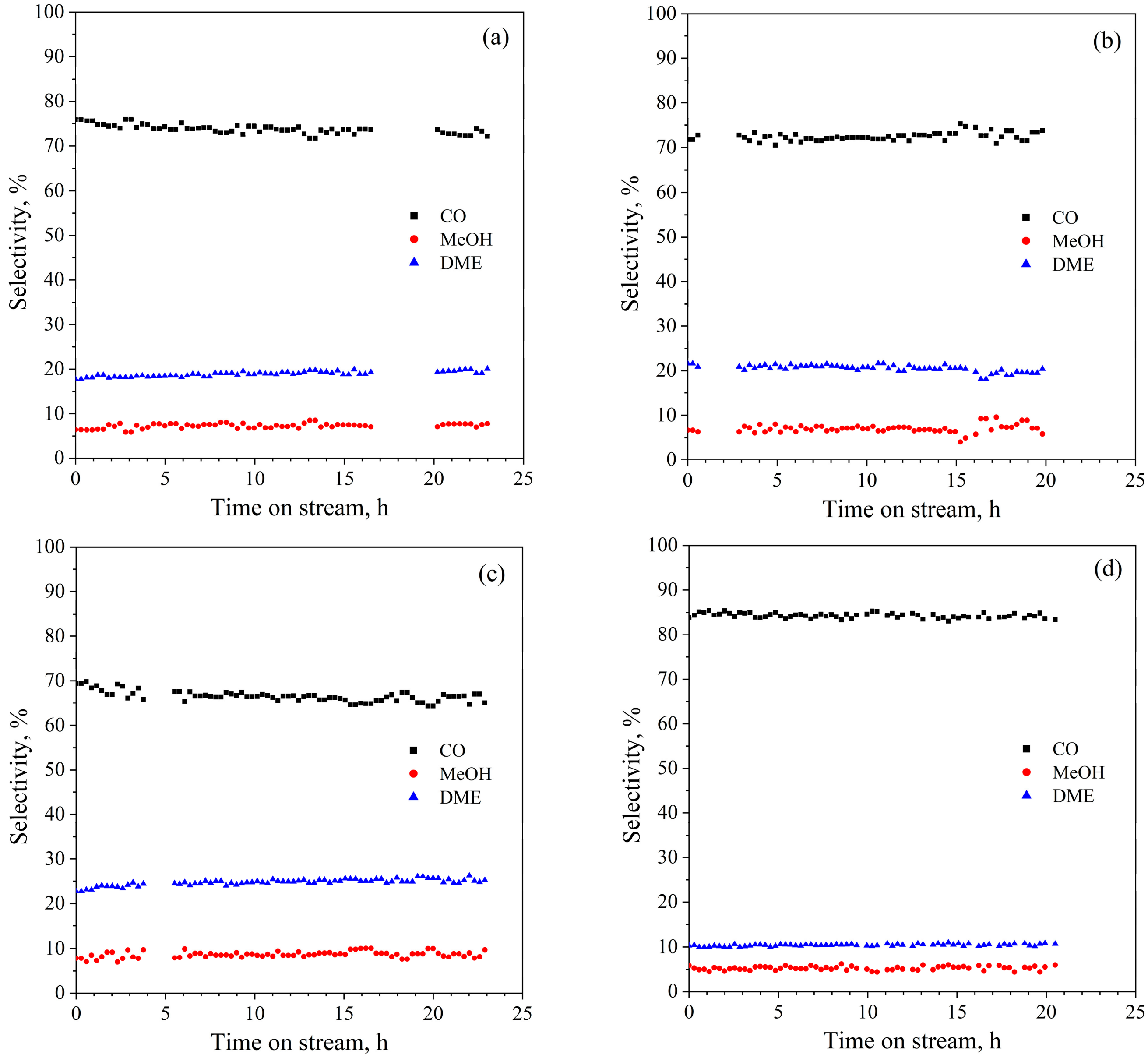

Figure S10. DME, MeOH and CO selectivity profiles of the hybrid catalysts during the stability test: (a) CZZ/FER OX 1:2, (b) CZZ/FER OX 2:1, (c) CZZ/FER WI 1:2 and (d) CZZ-FER MIX 1:2. Reaction conditions: 2.5 MPa; 275 °C; 13.3 $NL \cdot g_{cat}^{-1} \cdot h^{-1}$; inlet $H_2/CO_2/N_2$, 3/1/1 mol/mol.
